# A Fact Stranger Than Fiction

**Published:** 1891-04

**Authors:** 


### A FACT STRANGER THAN FICTION.

This story of a lost man rescued, and a starving baby saved, is told by a Texas traveller : I checked my horse and after one long, straining look around owned to myself that I was lost. I had suspected the fact some time, but had stubbornly fought down the suspicion, though my horse evidently realized it. With patient endurance he plodded along, resignation plainly expressed in the droop of his tail and ears. In place of the ranch, the hearty welcome, pleasant words, bed, supper, and fire I had expected to reach by sunset, there was nothing to be seen before, behind, on either hand, but the dead level of the plain. There were paths in plenty ; in fact, the trouble was there were too many — all narrow and winding, for whose meandering there seemed not the slightest excuse, except the general tendency to crookedness most things, animate and inanimate alike possess. But it would have taken the instinct of a bloodhound or a trailing Indian to have said which paths had been made by horses' feet or those of cattle.

Now that the sun was gone, I found my knowledge of the point of the compass gone with it. As I sat perplexed and worried, the gloom of twilight gathered fast and the chill of coming rain smote me through and through, while in the distance there was the roll of thunder. Glancing up I saw that the masses of cloud had closed together in a curtain of gray mist. My horse strode on of his own accord, and hoping that his instinct would lead us to some house, I let him have his will. Presently it began to rain, a sort of heart-broken passionless weeping, but with a steady determination to persevere all night, that awoke graver apprehension in my bosom than any amount of blustering, showery downpour could have done. This fine, still rain was accompanied by a low, soughing wind that added its desolate note to the general dreariness of the hour. Of course, I did not mind a little rain, but the prospect of spending the entire night exposed to it was any thing but agreeable, and I grew really violent in denunciation of the folly which had led me, an utter stranger in the country, to attempt to find any thing less than a volcano in active eruption on a bald prairie.

The Texans are a fine people, in many respects the most admirable of hosts—but individually and collectively they lack any appreciation of distance. This is due, of course, to their having so much space around them ; but to a stranger ignorant of the extent to which the phrases "a little piece out," and "just outside o' town," can be stretched, this contemptuous regard of miles is a little misleading. But in the face of that dreary, monotonous moaning of rain and wind, even my anger at my own folly could not burn long, and though chilled to the bone and tired and hungry, I plodded on dully, grateful that no night, even the longest, could last forever. It was now quite dark, and very dark at that, though at short intervals close to the horizon a faint gleam of lightning showed, too distant to cast brightness on my path and only sufficient to intensify the blackness about me.

All at once I saw a man walking about fifteen feet in front of me. Yes, I know I said it was intensely dark, but all the same, I repeat it, I saw a man walking in front of me, and furthermore I could see that he was a large man, dressed in rough, but well-fitting clothes ; that he wore a heavy red beard, and that he looked back at me from time to time with an expression of keen anxiety on his otherwise rather fixed features.

" Hallo !" I cried, but as he did not halt, I concluded he did not hear me. As a second hail produced no result I spurred my weary horse up to overtake the stranger. But, though the gray responded with an alacrity most commendable under the circumstances, I soon found that this strange pedestrian did not intend to let me catch up with him. Not that he hurried himself. He seemed without any exertion to keep a good fifteen feet between us. Then I began to wonder how, with the intense darkness shutting me in as four black walls, I was yet able to see my strange companion so clearly, to take in the details of his dress, and even the expression of his face, and that at a distance more than twice my horse's length, when I could hardly see his head before me. I am not given to superstitious fancies, and my only feeling was of curiosity.

We went on in silence for nearly half an hour, when, as suddenly as he had appeared, he was gone. I looked around for him, half afraid, from his instant and complete disappearance, that I had been dreaming, when I perceived that I was close to a small, low building of some sort. I reined in and shouted several times, but not the slightest response could I hear, and at last I rode boldly up and tapped on the wall with the butt of my riding whip. Then, as this elicited no sign of life, I concluded that I had stumbled on some deserted house, or that it was the abode of my eccentric friend ; so, dismounting and tying the gray, I resolved to spend the rest of the night under a roof or to find some good reason for continuing my journey. I felt my way along the wall till I reached a door, and, trying this and finding that it yielded to me, I stepped inside, striking a match as I did so. Fortunately, I carried my matches in an air-tight case, and as it was dry the one I struck gave me a light at once. I found myself in a large room close to a fireplace, over which a rude shelf was placed, and on this mantel I saw an oil lamp, to which I applied my match.

On the hearth was heaped a quantity of ashes and over these crouched a child, a little girl of 5 or 6. At the other end of the room which was plainly and scantily furnished, lay a man across a bed, and as I raised the lamp I saw that he was the same I had been following, but there was something in his attitude and face that struck me as peculiar, and I was about to go forward and look at him, when the child who had at first seemed dazed at the light fairly threw herself upon me.

"Have you any thing for Nelly to eat ?" she said, and then : "Oh, Nelly so hungry !"

I ran my hand into my pocket and drew forth what had been a paper bag of chocolate candy, but was now a pulpy unappetizing mass. I must confess to a childish fondness for sweets, which I usually carry in some form about me. I handed the remains of my day's supply to the child, and then walked over to the bed. Yes, it was the same man, red beard, rough clothes, but setting off the magnificent frame to perfection ; the same man, but dead, long dead.

I took his hand only to find it stiff and cold, while his face had the dull gray aspect never seen in the newly dead. As I stood gazing down on him a little hand touched mine.

"Nelly so hungry !" said the child.

"Have you eaten all the candy ?" I asked her.

"Yes, yes ! But me hungry, for me had no dinner, no brekkus, no supper, and papa won't get up."

The house, which consisted of the large room, a smaller kitchen, and a shed, where I found a quantity of hay and fodder, seemed quite bare of food, but by dint of searching in the hay I discovered a nest, which Nelly informed me was there, and in it two fresh eggs. These I boiled for her. When she had finished I soothed her to sleep on a bed I made for her before the fire. Then after I had put my horse in the shed room and fed him, I performed as well as I could a service for the dead.

When day dawned I was able to discern at some distance from the house a line of telegraph poles, and taking the child with me I followed these to the nearest town, where I notified the authorities of the death.

The dead man's name was Frederick Barnstaple. He was an Englishman, so I found, a recent arrival in those parts. His daughter was restored to her family across the water, and is now a pretty girl of 17. I have never told this story before, but I am ready to take an affidavit to its truth. It all happened about thirty miles from Dallas.

